# Supplementary material for: Solution-Grown MAPbBr3 Single Crystals for Self-Powered Detection of X-rays with High Energies above One Megaelectron Volt
Source: Nanomaterials (Basel). 2023 Jul 25;13(15):2157. doi: 10.3390/nano13152157 (PMC10421116; doi:10.3390/nano13152157)
Supplement: Supplementary file 1 [file nanomaterials-13-02157-s001.zip › nanomaterials-2523977-supplementary.pdf]

Supporting Information

Solution-Grown $MAPbBr_3$ Single Crystals for Self-Powered Detection of X-rays with High Energies above One Megaelectron Volt

Beomjun Park [1,2], Juyoung Ko [1], Jangwon Byun [1], Sandeep Pandey [1,2], Byungdo Park [3], Jeongho Kim [3] and Man-Jong Lee [1,2,*]

[1] Department of Chemistry, Konkuk University, Seoul 05029, Republic of Korea
[2] Advanced Crystal Material/Device Research Center, Konkuk University, Seoul 05029, Republic of Korea
[3] Department of Radiation Oncology, Samsung Changwon Hospital, Sungkyunkwan University School of Medicine, Changwon 51353, Republic of Korea
* Correspondence: leemtx@konkuk.ac.kr

Supplementary Note S1. Dark current data

$$Current_{(t)} = \begin{cases} 1 \pm \varepsilon \ (before\ RI) \\ 3 \pm \varepsilon \ (after\ RI) \end{cases}, \tag{S1}$$

where RI and ε are radiation illumination and errors depending on its distribution, respectively. In Fig. 3a, average dark current was set to the normalized value of 1 at both detectors. The distribution of $current_{(t)}$ in ‘Low ρ detector’ was set to 5 % of dark current, while that in ‘High ρ detector’ was 1 %. When both detectors absorb incoming radiation, generating photo-current (ΔI) was set equally to 2. ΔI can be defined as;

$$\Delta I = I_{on} - I_{off}, \tag{S2}$$

where, I_{on} and I_{off} mean the current under radiation illumination or not. Data formation was simulated with Matlab R2020b.

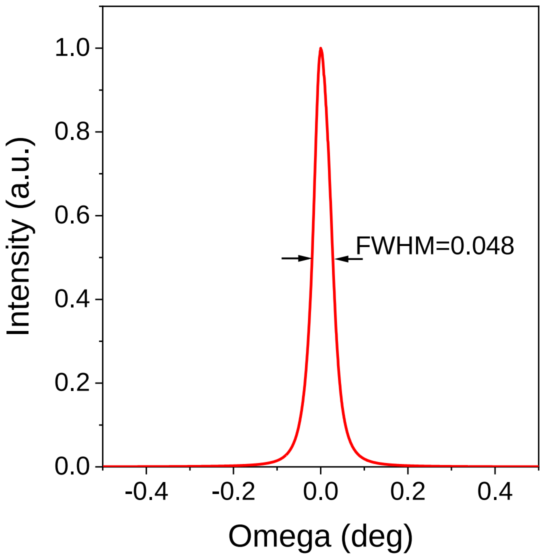

Figure S1. XRD rocking curve of (100) peak in $MAPbBr_3$ single crystal.

Supplementary Note S2. Drift current density

Drift current density (J_d) consists of electron drift current density (J_{de}) and hole drift current density (J_{dh}) (Eq. S3) under an electric field (E).

$$J_d = J_{de} + J_{dh} = qn\mu_e E + qp\mu_h E = qE(n\mu_e + p\mu_h) \quad \text{(S3)}$$

The q value is the standard charge (1.6×10^{-19} C). The n and p values are charge carrier density, and μ is the mobility of each carrier. When introducing the different electric field (E_1 or E_2), different drift current density can be induced despite the same carrier density (n or p) and transport property (μ_e or μ_h).

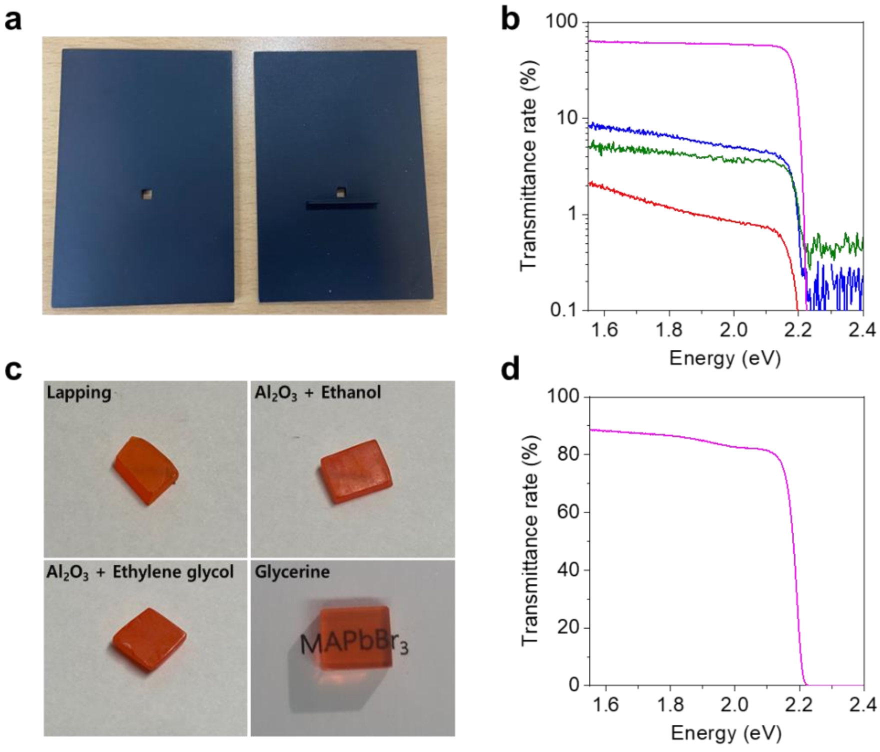

Figure S2. Data for identifying transmittance property of MSCs. (a) the 3D-printed plate for normalization of input photons' intensity in UV-visible measurements. (b) Transmittance rate of MSCs depending on the abrasive, which was reformed to log-scaled Y axis. (c) Photographs of MSCs depending on the abrasives, and the letters "$MAPbBr_3$" were written on the paper under each sample. (d) Transmittance rate of the glycerin-polished MSC in small region with normalized plate of vendor (part No.0410204100, Aperture mask, solid sample holder, 1mm). The higher transmittance rate is observed.

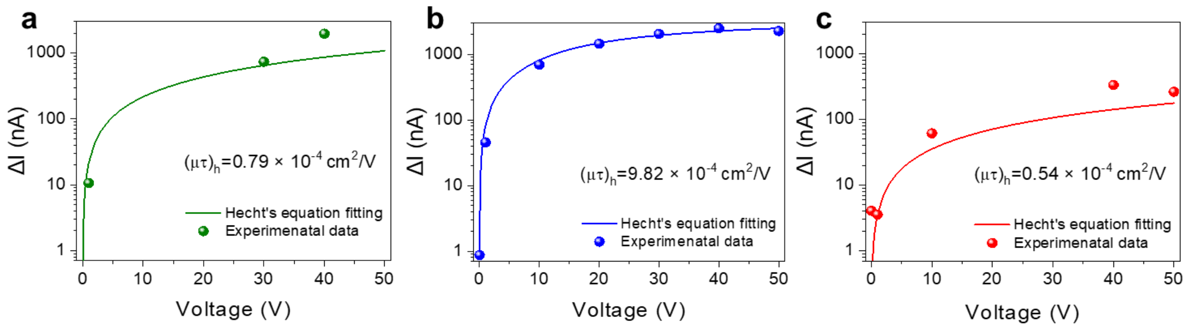

Figure S3. Transport properties of MSC depending on the polishing abrasives. Collected current under radiation source and their Hechts' equation fittings of each processed MSC; (a) lapping, (b) Al_2O_3+ethanol polishing, and (c) Al_2O_3+ethylene glycol polishing.

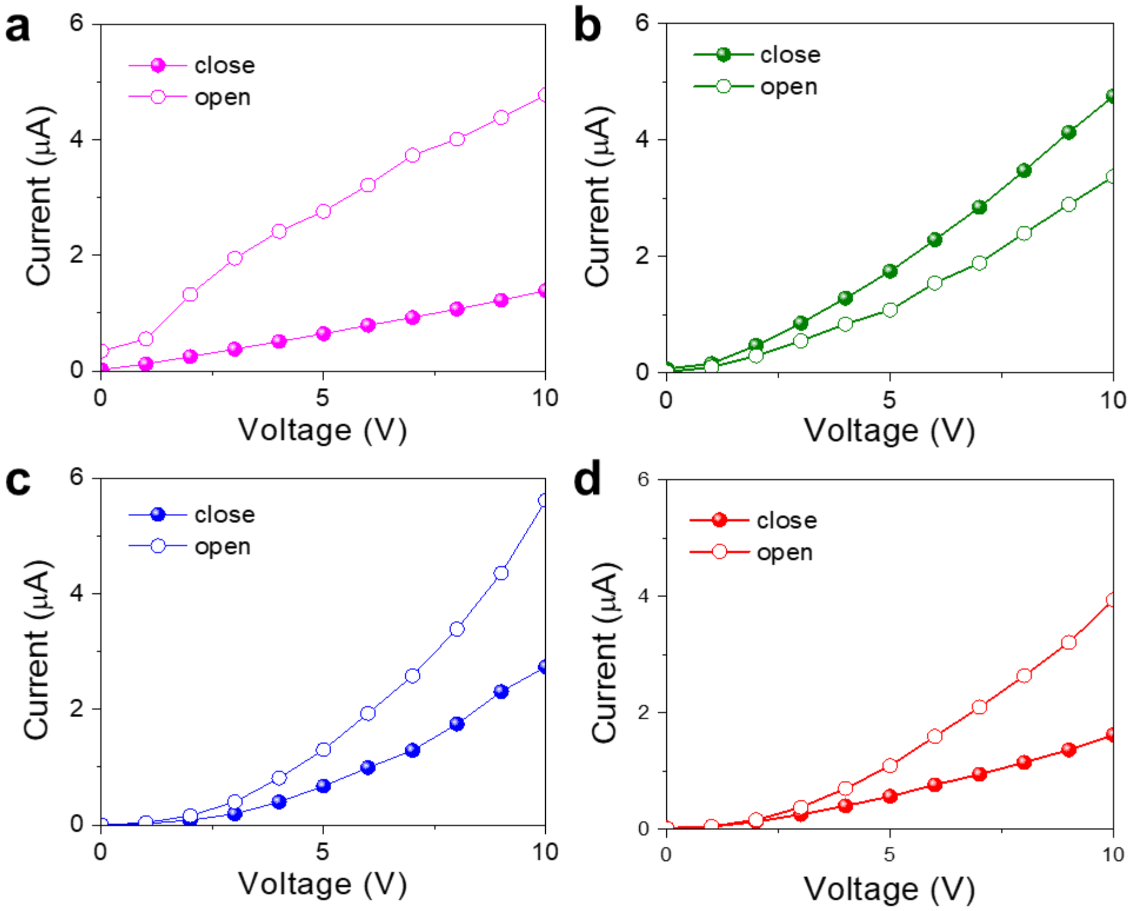

Figure S4. Photo-current of each MSC with/without the light from fluorescence lamp. The processes of each MSC are, in order, (a) glycerin, (b) lapping, (c) Al_2O_3+ethanol polishing, or (d) Al_2O_3+ethylene glycol polishing.

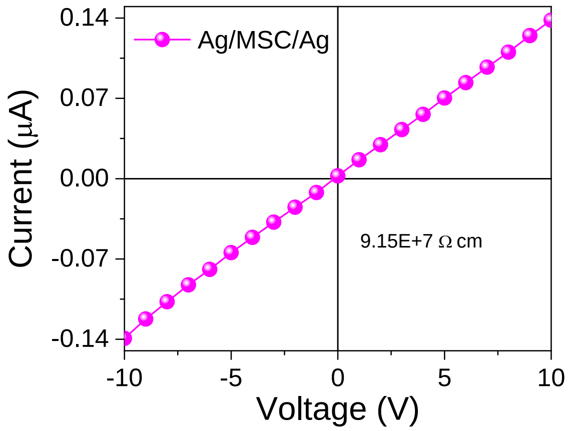

Figure S5. I-V curve of Ag/MSC/Ag polished with glycerin under the dark box.

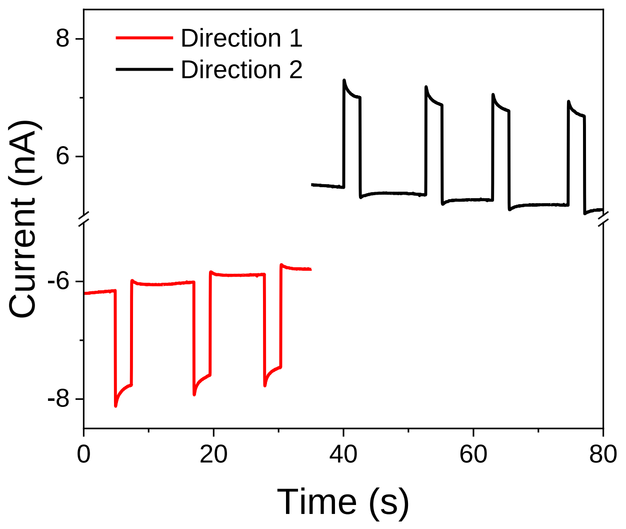

Figure S6. Photocurrent signals of 70 kVp X-rays under zero bias depending on the direction.

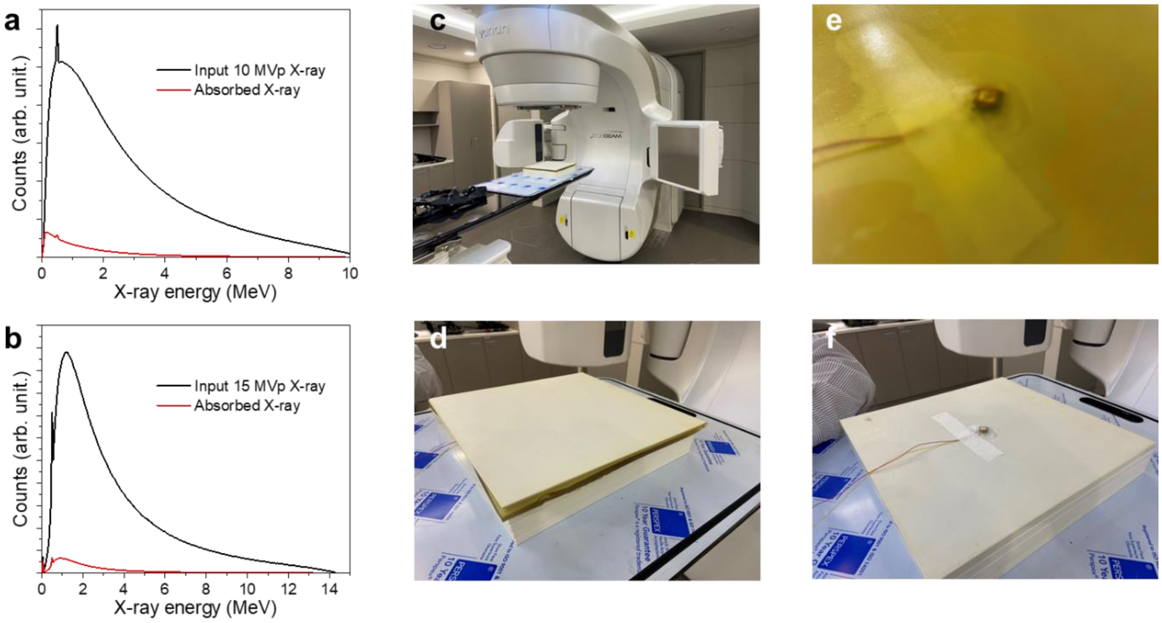

Figure S7. Simulation and experimental conditions of high energy X-ray detection with 2 mm MSC. (a, b) The served input spectra and their absorbed spectra by 2 mm MSC. (c) Linear accelerator (TrueBeam; Varian Medical Systems) used as high energy X-ray source. (d) Geometry for high energy X-ray detection. (e, f) The condition inside of the phantoms. The proper thicknesses of the soft tissue equivalent phantom are 1.4, 2.3, and 2.8 cm at 6, 10, and 15 MVp X-rays, respectively.

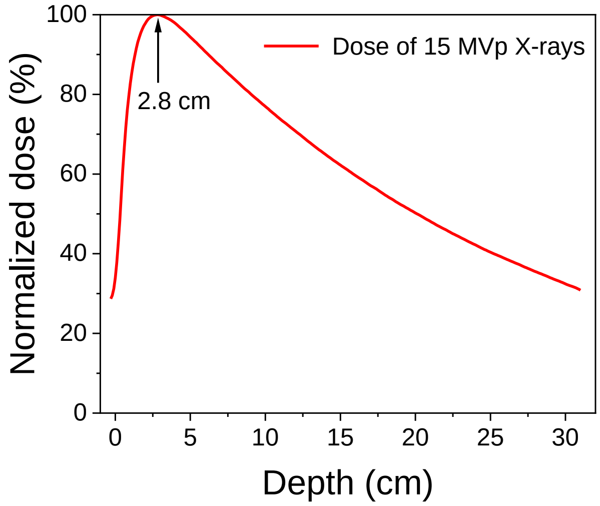

Figure S8. The measured percentage depth dose (PDD) of TrueBeam used in this study. The 15 MVp X-rays and 10×10 cm^2 of field size were applied.

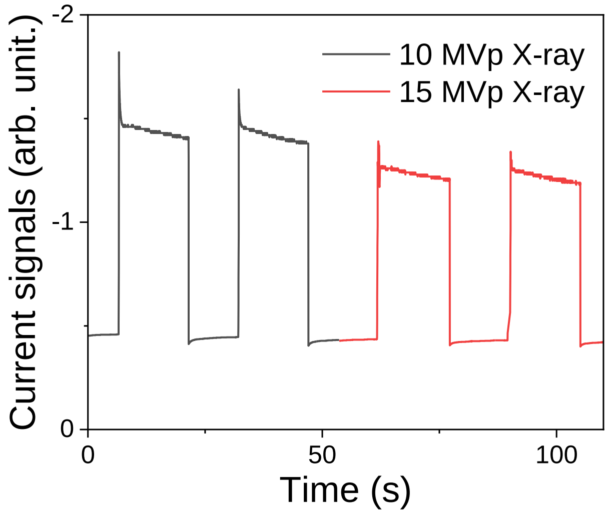

Figure S9. Photo-current signal from high energy X-ray sources based on the processed MSC detector.

Supplementary Note S3. Monitor unit (MU)

Monitor unit (MU) has been used in the radiation therapy field to standardize the dose unit of radiotherapy equipment in the world. When 100 MU applies, 1 Gy of the absorbed dose is transferred to the maximum dose point in Fig. S10.
